# Quantifying the connections—linkages between land-use and water in the Kathmandu Valley, Nepal

**DOI:** 10.1007/s10661-018-6687-2

**Published:** 2018-04-23

**Authors:** Jeffrey C. Davids, Martine M. Rutten, Ram Devi T. Shah, Deep N. Shah, Nischal Devkota, Petra Izeboud, Anusha Pandey, Nick van de Giesen

**Affiliations:** 1 Water Management, Delft University of Technology, TU Delft Building 23, Stevinweg 1, 2628 Delft, CN Netherlands; 2 SmartPhones4Water, Chico, USA; 3 Aquatic Ecology Center, Kathmandu University, Dhulikhel, Nepal; 4 Himalayan Biodiversity and Climate Center (HimBioCliC), Bhaktapur, 44800 Nepal; 5 Central Department of Environmental Science, Tribhuvan University, Kirtipur, Nepal; 6 SmartPhones4Water-Nepal (S4W-Nepal), Thusikhel, Lalitpur, Nepal; 7 Environmental Science, Nayaa Aayaam Multi-Disciplinary Institute (NAMI), Jorpati, Nepal

**Keywords:** Land-use, Water quality, Kathmandu Valley, Land-water linkages, Rapid stream assessment (RSA)

## Abstract

Land development without thoughtful water supply planning can lead to unsustainability. In practice, management of our lands and waters is often unintegrated. We present new land-use, ecological stream health, water quality, and streamflow data from nine perennial watersheds in the Kathmandu Valley, Nepal, in the 2016 monsoon (i.e., August and September) and 2017 pre-monsoon (i.e., April and May) periods. Our goal was to improve understanding of the longitudinal linkages between land-use and water. At a total of 38 locations, the Rapid Stream Assessment (RSA) protocol was used to characterize stream ecology, basic water quality parameters were collected with a handheld WTW multi-parameter meter, and stream flow was measured with a SonTek FlowTracker Acoustic Doppler Velocimeter. A pixel-based supervised classification method was used to create a 30-m gridded land use coverage from a Landsat 8 image scene captured in the fall of 2015. Our results indicated that land-use had a statistically significant impact on water quality, with built land-uses (high and low) having the greatest influence. Upstream locations of six of the nine watersheds investigated had near natural status (i.e., river quality class (RQC) 1) and water could be used for all purposes (after standard treatments as required). However, downstream RSA measurements for all nine watersheds had RQC 5 (i.e., most highly impaired). Generally, water quality deteriorated from monsoon 2016 to pre-monsoon 2017. Our findings reinforce the importance of integrated land and water management and highlight the urgency of addressing waste management issues in the Kathmandu Valley.

**Electronic supplementary material:**

The online version of this article (10.1007/s10661-018-6687-2) contains supplementary material, which is available to authorized users.

## Background and introduction

### Land-use-water linkages

Many studies have highlighted the strong linkages between land-use and water resources, from both process and planning perspectives (Pereira 1973; Ghassemi et al. 1995; Calder 1999; Tong and Chen 2002; Mitchell 2005; Wilson 2015; Mirzaei et al. 2016; Li et al. 2017; and others). Yet, in many parts of the world, land-use planning and water resource management continue to be implemented in an unintegrated fashion (Foley et al. 2005). This situation is frequently exacerbated in developing countries by a combination of weak political and financial institutions, deficient physical infrastructure, and limited understanding of the physical processes that link the two. The resulting sum of a series of economically or politically sensible land-use planning decisions often leads to intractable water management predicaments (Shah et al. 2003; Scott and Shah 2004; Harou and Lund 2008). Additionally, the data necessary to analyze changes in both land-use and water quality and quantity over space and time are often not available (Gleick 1998; Hannah et al. 2011; Shrestha et al. 2012; Van de Giesen et al. 2014; and others).

Several examples exist the world round, of rapid and largely unplanned urban growth completely outpacing necessary freshwater delivery and waste water treatment infrastructures (Girija et al. 2007; Ramachandraiah and Prasad 2004; Du et al. 2010; Shah and Shah 2013; Carley and Christie 2017). This eventually leads to degradation of surface water and groundwater, including dependent ecosystem services (Carpenter et al. 1998; Ellis 1999; Regmi et al. 2017). The primary factors leading to this degradation include direct discharge of untreated urban and industrial effluents (nitrogen, solvents, fecal contaminants, etc.) and uncontrolled agricultural waste discharges (e.g., nitrogen, phosphorus, pesticides, salt, etc.).

### Kathmandu Valley

**Fig. 1:**
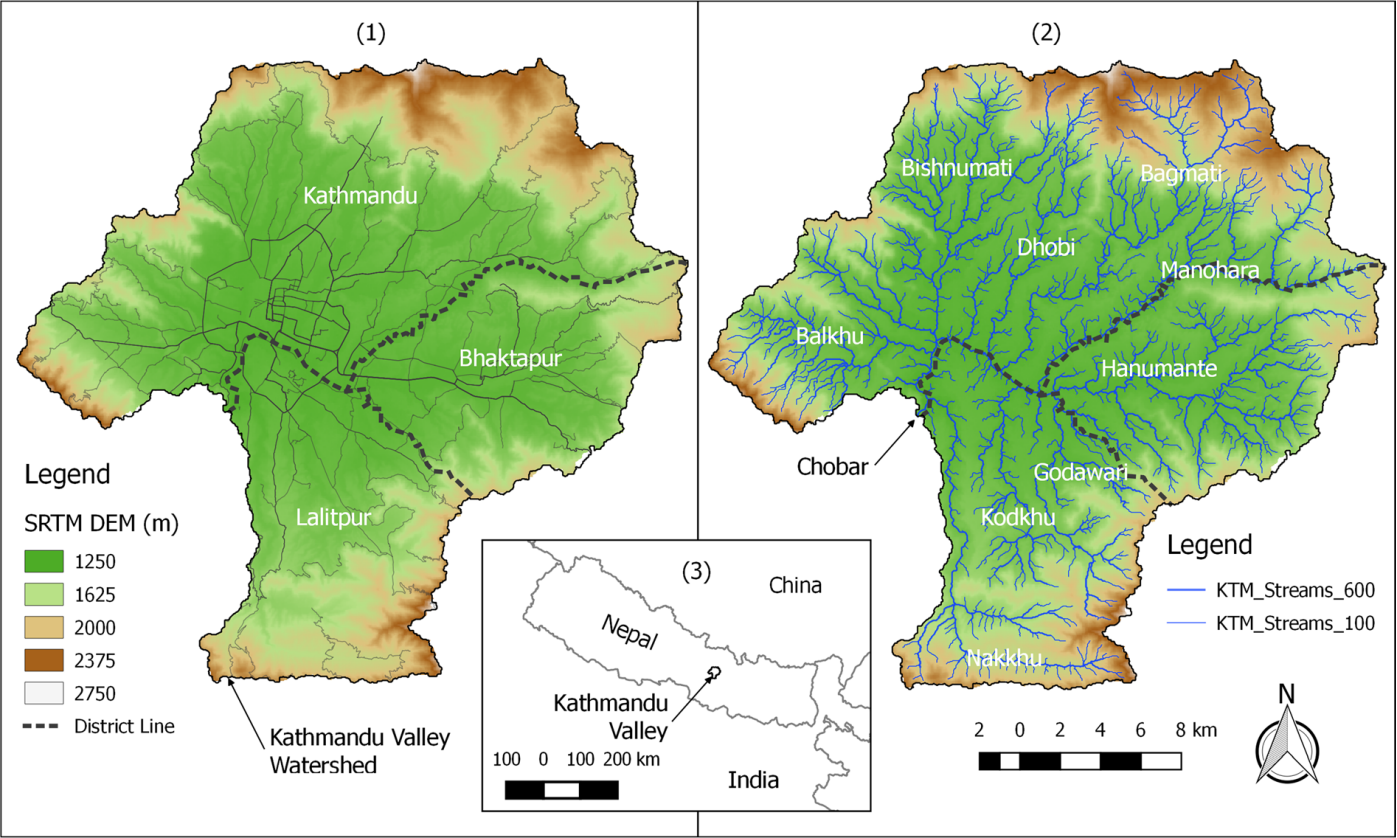
Kathmandu Valley Watershed with roads, district boundaries, and SRTM DEM at 30-m resolution (1), stream network with nine perennial streams labeled (2), and location map of Nepal and the Kathmandu Valley (3). The Kathmandu Valley Watershed shown uses Chobar as the pour point

The Kathmandu Valley (Valley; Fig. 1) is a small intermontane basin roughly 25 km in diameter with a total land area of 587 km^2^ in the Central Region of Nepal. Population in the Valley has increased significantly in the last 25 years (Thapa et al. 2017), with official estimates of somewhere between 2.2 and 2.5 million people living in one of the three major districts of Kathmandu, Lalitpur, and Bhaktapur (CBS 2011, ISRC 2008, World Bank 2013). Once a lacustrine environment, the Valley floor has a generally mild southerly slope and contains relatively deep and fertile deposits of gravels, sands, silts, and clays, from north to south (Shrestha et al. 1999; Shrestha et al. 2012). These soils, and increasingly the underlying groundwater system, support wide-scale agriculture within the Valley, consisting primarily of rice, corn, vegetables, and other cereals.

The Valley is principally drained by the Bagmati River, whose headwaters originate at the perennial springs on the southeastern slopes of Shivapuri Peak. Eight other historically perennial tributaries join the Bagmati River, prior to it exiting the southwestern edge of the Valley near Chobar. Elevations in the Valley range from 1260 m near Chobar, to 2780 m at Phulchowki Peak, the headwaters of the Godawari River. Precipitation patterns are dominated by the South Asian monsoon, with 80% of precipitation occurring between June and September (Shrestha 2000). Due to the topography of the Valley, and the strong south to north monsoonal air movement, there are large precipitation gradients from rain shadow and orographic effects on the southern and northern portions of the Valley, respectively.

Due in part to a lack of integrated land-use and water resources planning, the Kathmandu Valley currently suffers from both water quantity and quality crises. Uncontrolled urban expansion into the fringes surrounding the historically populated areas is increasing demand for water, intensifying discharge of untreated wastewater discharged to streams, and reducing recharge potential for the progressively stressed underlying aquifer system (Shrestha et al. 2012). We searched for pertinent literature that characterizes these issues using Google Scholar and the key search terms *land-use*, *water*, *management*, *quality*, *quantity*, and *Kathmandu*.

Regarding land-use, Rimal (2011) found that the area of built land (i.e., urban, industrial, etc.) within the core of the Kathmandu Metropolitan area increased nearly fourfold (i.e., 395%) in just over three decades from 1976 to 2009. Within the hill regions of Nepal, Paudel et al. (2016) found that urban land uses were increasing rapidly in the Kathmandu and Pokhara Valleys. Uddin et al. (2015) developed a land cover map for the entire country of Nepal for 2010. However, possible land-use changes between 2010 and 2016 (i.e., when the field work was performed) reinforced the need for an updated land-use coverage focus on the Kathmandu Valley.

Several studies have highlighted the degradation of water quality in the Valley, with many of them focusing on groundwater quality, since it is a critical water supply (Khadka 1993; Chettri and Smith 1995; Jha et al. 1995; Ha and Pokhrel 2001; Merz et al. 2003; Kannel et al. 2007; Shah et al. 2008; Shrestha et al. 2012; Shrestha et al. 2014; Shrestha et al. 2017; Haramoto 2018). Shrestha et al. (2008) mapped the water quality of the Bagmati River in the Kathmandu Valley and found that water quality was extremely poor in rivers sections inside built areas, fair in agricultural dominated areas, and good in most upper stretches of the rivers which are generally forested and inside protected areas. In the meantime, biological methods have been developed and evaluated for integrated measurement of the status of water quality in rivers (e.g., Hartmann et al. 2010; Shah and Shah 2012). Shah and Shah (2013) presented benthic macroinvertebrate assemblage as an indicator of ecological status along the Bagmati River and a few tributaries in the Kathmandu Valley. While they did not quantify tributary land-use composition, they did conclude that benthic macroinvertebrate assemblages reflected the actual ecological status and they observed changes between seasons at the studied sites. Finally, by performing a baseline study along the Bagmati to collect physical, chemical, and biological indicator data regarding water quality and water pollution, Milner et al. (2015) found that pollution originating from the Kathmandu Valley persisted to 75 km downstream from Chobar (i.e., the outlet of the Bagmati River from the Kathmandu Valley).

While our literature review showed that several studies have focused on land-use changes or water quality in the Kathmandu Valley, we could not identify any quantitative assessments of the impacts of land-use on water quality and quantity. Therefore, the goal of this paper is to improve understanding of the longitudinal (i.e., upstream to downstream) linkages between land-use and water quality and quantity for both monsoon and pre-monsoon periods in the Kathmandu Valley. We do this by collecting, analyzing, and presenting new land-use, ecological stream health, water quality, and stream flow data from the perennial tributaries to the Bagmati River in the Kathmandu Valley (Valley), Nepal.

## Materials and methods

To better understand the impacts of land-use on water in the pre-monsoon and monsoon periods, we first delineated the locations of streams in the Kathmandu Valley. Next, we collected new field data including streamflow, basic water quality, rapid stream assessments, and land-use ground observations. Then, we developed a land-use coverage and watershed delineations for each of our stream measurement locations. We then used the combination of these field and derived geospatial data to visually represent how water quality and quantity changed as a function of land-use. Finally, we performed a correlation analysis to quantify these relationships.

### Stream network generation

Using Quantum Geographic Information System (QGIS) as a user interface, we used the Geographic Resources Analysis Support System (GRASS) module r.watershed to develop a stream network for the Kathmandu Valley. First, a Shuttle Radar Telemetry Mission (SRTM) 30-m digital elevation model (DEM) was used to create a raster coverage of drainage directions between each pixel and the surrounding eight pixels (SRTM 2000). Then, an accumulation raster was developed, where the number of upstream pixels draining to each pixel was quantified. Finally, thresholds of 100 and 600 upstream pixels were used to create both a fine and course scale stream network raster, which was converted to a vector coverage. These and other Python scripts can be found in the following GitHub repository: https://github.com/jcdavids/KathmanduLandUseWater.

### Field data collection

For each of the nine perennial watersheds in the Valley, we identified between three and seven locations for performing the field data collection activities described below. Emphasis was placed on performing upstream measurements prior to considerable non-natural land-uses and downstream measurements near the confluence with other tributaries. All field measurements were collected digitally in the field with an Android application called Open Data Kit or ODK (Anokwa et al. 2009). ODK was used to record GPS coordinates and take photographic documentation for all observations.

**Fig. 2:**
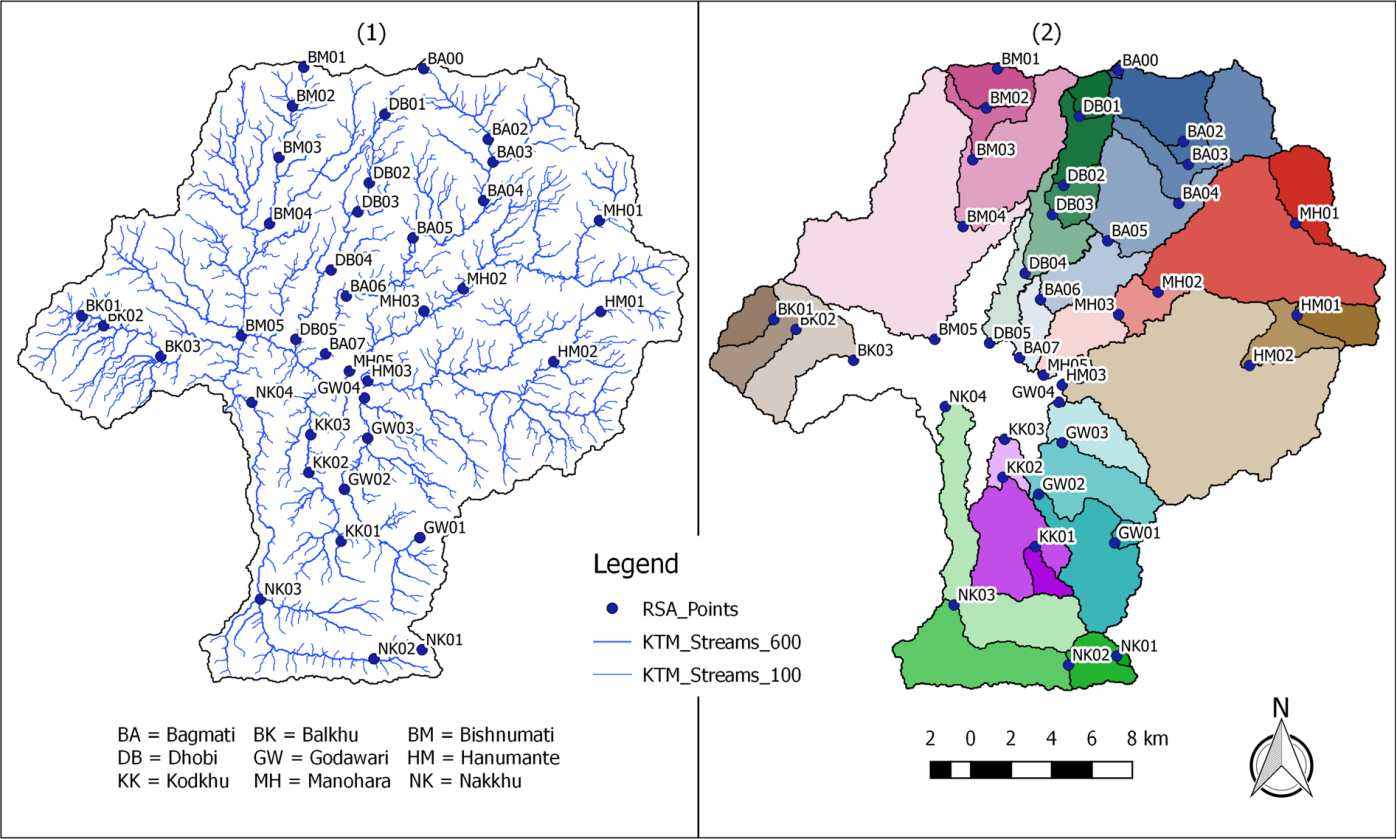
Thirty-eight measurement locations within the Kathmandu Valley (1) and resulting upstream watersheds for each location (2)

Field data collection was performed in two different periods to characterize both monsoon and pre-monsoon conditions. Monsoon sampling was performed from the 5th to the 30th of September 2016. Except for one measurement (i.e., BA00; see Fig. 2 for details), pre-monsoon sampling was performed between the 18th of April and the 17th of May 2017. Efforts were made to use the same personnel and equipment for both monsoon and pre-monsoon assessment to ensure data compatibility. Additionally, during pre-monsoon sampling, care was taken to avoid sampling during or after precipitation events, to ensure that measurements were representative of baseflow or near baseflow conditions. In practice, this meant that field work was stopped if runoff generating rainfall events occurred. Sampling was later resumed when water levels returned to pre-event levels.

#### Streamflow measurements

We measured streamflow at all locations with a SonTek FlowTracker Acoustic Doppler Velocimeter (ADV) using the US Geological Survey (USGS) mid-section discharge method (Rantz 1982).

#### Basic water quality

We used a MultiLine® Multi 3630 IDS [WTW Germany] multiparameter meter to perform in situ measurements of temperature (T), electrical conductivity (EC), dissolved oxygen (DO), and (pH). Due to equipment problems, 2017 pre-monsoon pH measurements were not performed and approximately half of the pre-monsoon dissolved oxygen measurements were analyzed at ENPHO labs in Kathmandu.

#### Rapid stream assessment

We used the Rapid Stream Assessment (RSA) for Himalayan streams (Hartmann et al. 2010; Shah and Shah 2013) to assess ecological stream health at each sampling location. RSA has been used as an integrated and robust method to assess ecological stream health for over 5 years. RSA utilizes four primary classification categories including (1) sensory, (2) ferro-sulfide reduction, (3) bacteria, fungi, and periphyton, and (4) macroinvertebrate composition. Sensory features evaluated include smell, non-natural debris and turbidity. Ferro-sulfide reduction is used as a proxy for high organic loadings associated with high biological oxygen demand (BOD) and the associated reduction of dissolved oxygen (DO). Certain bacteria, fungi, and periphyton are indicators of the presence and/or absence of certain pollutants. Finally, macroinvertebrates’ richness and dominance of sensitive or tolerant organisms serve as a robust and integrated indicator of ecological stream health.

The output of the RSA process is a river quality class (RQC), ranging from one (1) to five (5), representing the best and worst quality rivers, respectively. RQC 1 represents natural to near natural waters suitable for all municipal, industrial, agricultural, and environmental purposes (after standard treatments as required). RQC 5, however, is most strongly impaired, with waters not suitable for any purposes. For each site, an RSA form was completed, georeferenced, and photographed via ODK.

#### Land-use ground observations

We used ODK to collect georeferenced photographic observations of land-uses at and around each RSA monitoring location. Land-use classes were based on the National Land Cover Database 1992 (NLCD92) introduced by the USGS Land Cover Institute (Vogelmann et al. 2001). Six land-uses classes were selected to represent the land-uses in the Kathmandu Valley: Forest; Shrubland, Agriculture Rice; Agriculture Non-Rice; Built Low; and Built High (Table 1). The sum of Forest and Shrubland are considered Natural land-uses; Agriculture Rice and Agriculture Non-Rice are collectively considered Agricultural land-uses; and Built Low and Built High are together consider Built land-uses. A total of 141 ground observations were recorded.

**Table 1:**
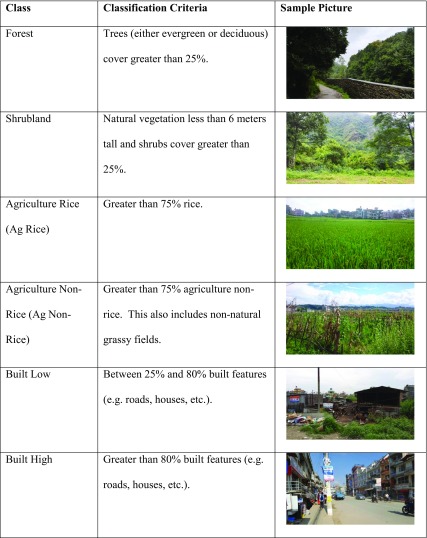
Description of land-use classes

### Land-use map and watershed delineations

#### Land-use map

We used the QGIS GRASS modules i.gensig and i.maxlik to assign per pixel a maximum likelihood for each land-use class. We performed this semi-supervised pixel based land-use classification on a cloud free Landsat 8 scene captured on October 7, 2015 (Gonzales et al. 2016). Two thirds of the 141 ground observations were used as training points for the spectral analysis algorithms, while one third were subsequently used as validation points of the resulting land-use map.

We assumed that land-use remained constant from the fall of 2015, when the Landsat image was taken, through the spring of 2017, when the pre-monsoon measurements were performed. Pre-monsoon sampling was performed prior to the planting of rice. Therefore, what was classified as rice in the October 2015 Landsat scene, was either weeds or bare earth being prepared for rice seedlings. Rice is usually planted roughly 2 to 4 weeks after when 2017 pre-monsoon sampling was completed.

#### Watershed delineations

With the drainage direction raster developed during the stream network generation process, we used r.water.outlet to determine the watershed delineation for each RSA monitoring point. Using the watershed delineations, the land-use coverage, and QGIS GRASS zonal statistics, we calculated the area of each land-use within each RSA watershed. We developed Python scripts with Matplotlib library to develop stacked area land-use proportion summaries with RQC, water quality, and water flow data plotted on the secondary vertical axes. These and other Python scripts can be found in the following GitHub repository: https://github.com/jcdavids/KathmanduLandUseWater.

### Correlation analysis

We used Pearson’s correlation coefficient r values (Lee Rodgers and Nicewander 1988) to characterize relationships between land-use and water quality and flow for 2016 monsoon and 2017 pre-monsoon data. The Pearson’s *r* values were tested for significance with a two-tailed *p* value hypothesis test.

## Results for the Kathmandu Valley

### Stream network, monitoring locations, and sub-watershed delineations

Figure 1 shows the original SRTM 30-m DEM (1) and the resulting stream network (2). The lighter and thinner blue lines represent streams with at least 100 upstream pixels. The darker and thicker blue lines represent streams with at least 600 upstream pixels. The Kathmandu Valley watershed boundary is shown, and uses Chobar as the pour point.

Figure 2 shows the 38 monitoring locations (1) and the resulting upstream watershed delineations for each location (2). A single color was chosen for each of the 9 tributaries, and the opacity was decreased from upstream to downstream. A tabular summary of the collected data is included in the supplementary data.

### Land-use coverage and land-use change figures

**Fig. 3:**
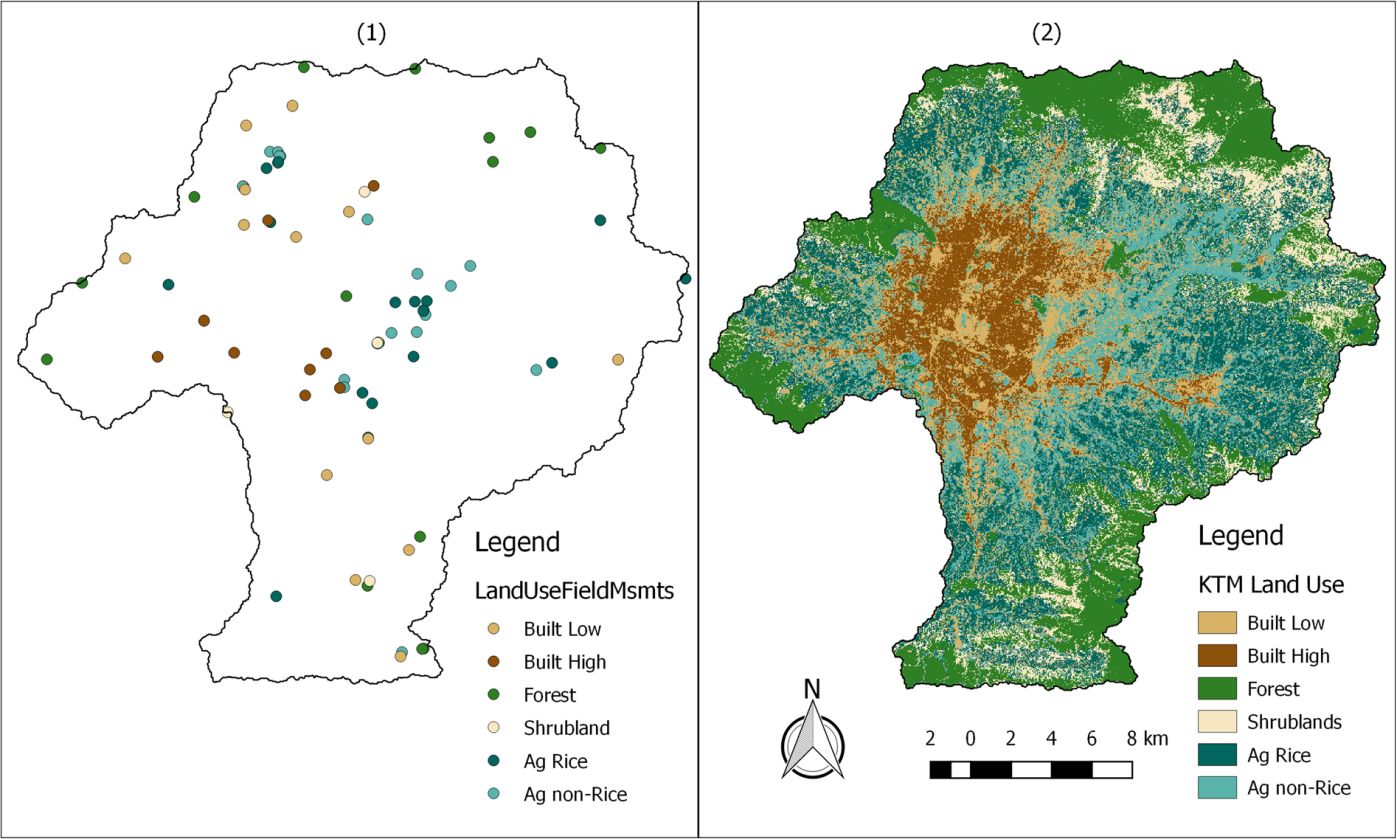
One hundred forty-one land-use observation points (1) and resulting 30-m resolution land-use classification map (2)

Figure 3 presents the locations of our 141 land-use observation points (1) and the resulting 30-m land-use raster coverage (2). 33% of the Valley was classified as natural land-uses comprised of 22% Forest and 11% Shrublands. Forty-one percent was classified as agriculture, with 24% Agriculture Rice and 17% Agriculture Non-Rice. The remaining 26% was classified as Built, with 16% low density, and 10% high density. There was an 88% agreement between the resulting land-use coverage and the land-use observations used for validation. For the remaining 12%, the disagreement was either small and explainable (i.e., a mix-up between high and low developed areas or between rice and non-rice agriculture), or was at points where the land-use classification on the ground was also in doubt. Detailed information about resulting land-use statistics can be found in the supplemental material.

**Fig. 4:**
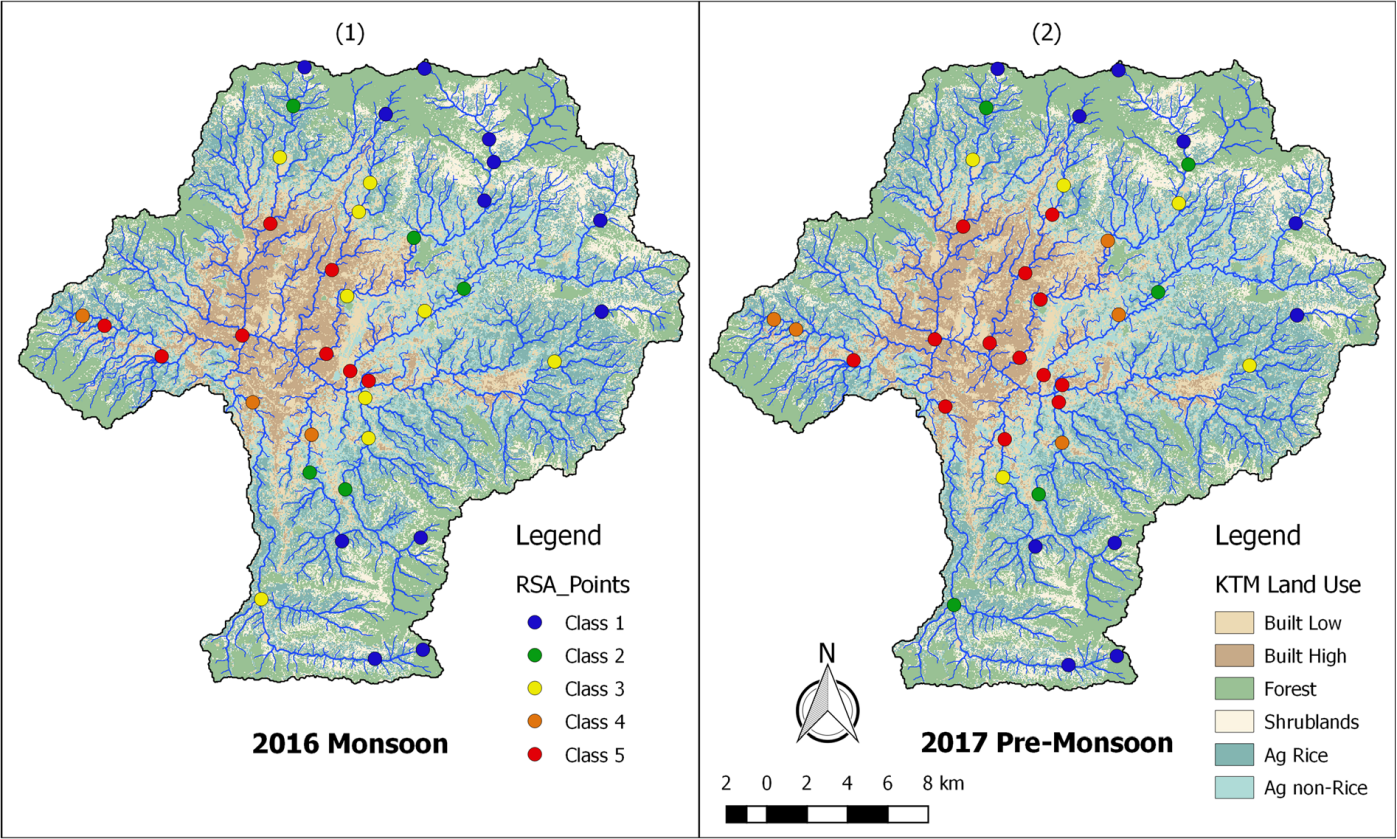
Map-based approach for both the 2016 monsoon (1) and 2017 pre-monsoon (2) data. Land-use colors are shown with 50% transparency to make RQC more viewable. Standard colors for RQC are used (Hartmann et al. 2010)

Figure 4 presents a map-based display of both the 2016 monsoon (1) and 2017 pre-monsoon (2) data. In both the monsoon and pre-monsoon data, locations with better ecological stream health were seen around the periphery of the Valley, with declining stream health moving toward the densely populated urban areas near the center of the Valley (shown in dark and light brown). Except for the Balkhu watershed to the west, the most upstream measurement of each watershed was either RQC 1 or 2 (i.e., blue or green). A noticeable upstream shift in RQC 4 and 5 (i.e., orange and red) was seen on the streams originating from the northern, eastern, and southern portions of the Valley. The Balkhu watershed to the west has the lowest overall ecological stream health for all three monitoring locations.

**Fig. 5:**
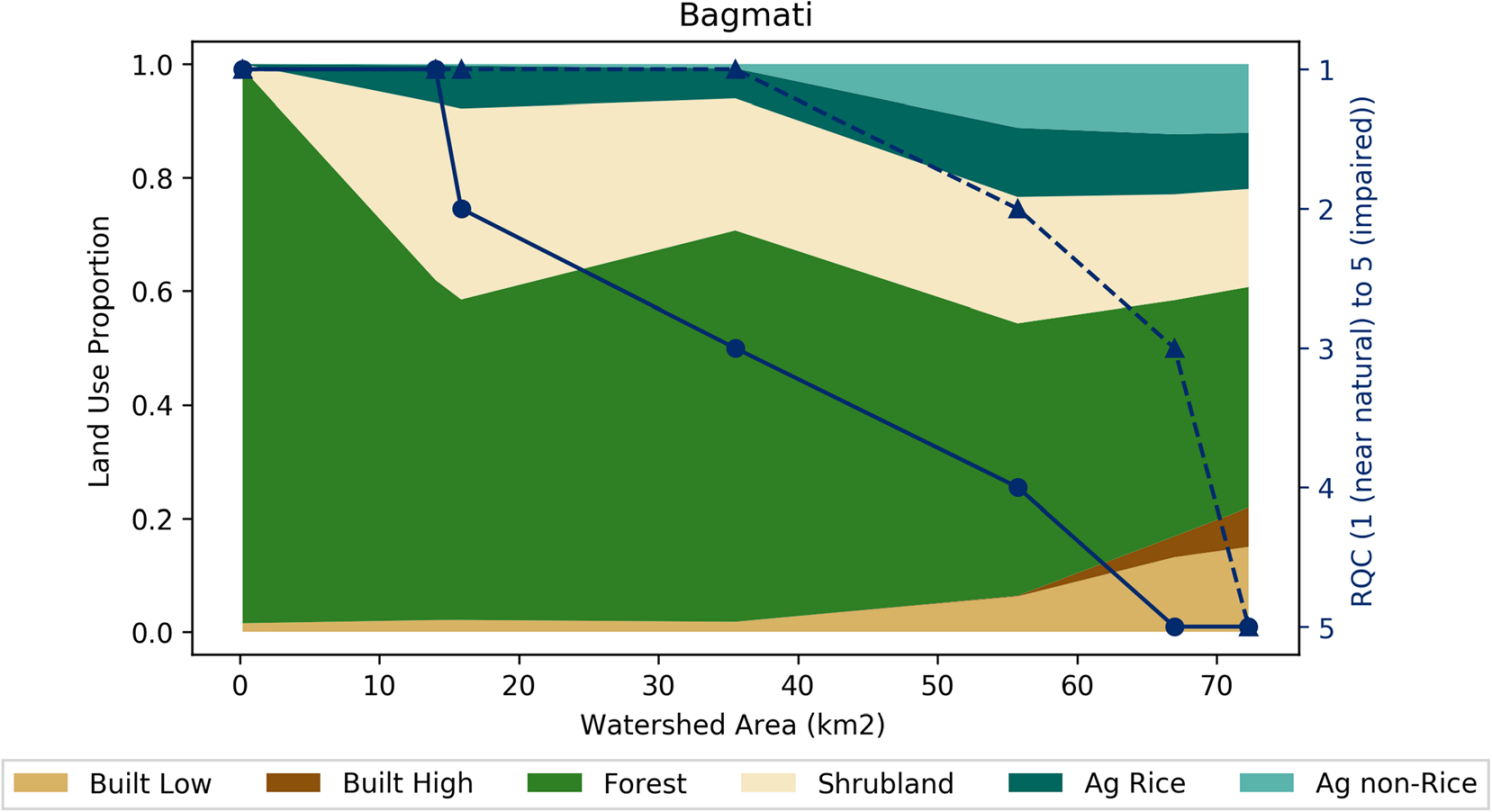
Land-use proportions and river quality class (RQC) for the Bagmati River in the Kathmandu Valley. Land-use proportions shown for six land-uses classes with reference to the primary (left) vertical axes. RQC shown for 2016 monsoon (dashed line with triangles) and 2017 pre-monsoon (solid line with circles) periods with reference to the secondary (right) vertical axes. The *x*-axis represents areas of the watersheds upstream of each measurement point, moving upstream to downstream (left to right). Watershed areas range from 0.2 to 72.3 km^2^. The six colors on the figure correspond with the six land-use classifications (Fig. 3). The relative vertical proportion of each color at each monitoring location represents the upstream proportion of each land-use (with reference to the primary (left) axis)

For the Bagmati River, RQC was determined at seven sites during the 2016 monsoon and 2017 pre-monsoon (Fig. 5; see supplemental materials for underlying data). The two upstream-most measurement sites were RQC 1 in both monsoon and pre-monsoon periods. RQC for the third through sixth sites diverges for monsoon and pre-monsoon. A deterioration in ecological stream health, illustrated by an increase in RQC, occurs from monsoon to pre-monsoon at all four of these sites. The fourth, fifth, and sixth sites showed a decline of two classes. The seventh and last site was RQC 5 for both the monsoon and pre-monsoon measurements.

**Fig. 6:**
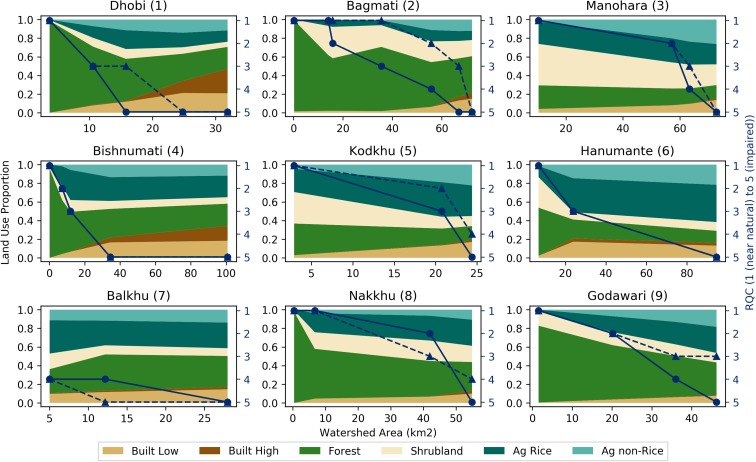
Land-use proportions and river quality class (RQC) for the nine perennial streams in the Kathmandu Valley. Land-use proportions shown for six land-uses classes with reference to the primary (left) vertical axes. RQC shown for 2016 monsoon (dashed line with triangles) and 2017 pre-monsoon (solid line with circles) periods with reference to the secondary (right) vertical axes

**Fig. 7:**
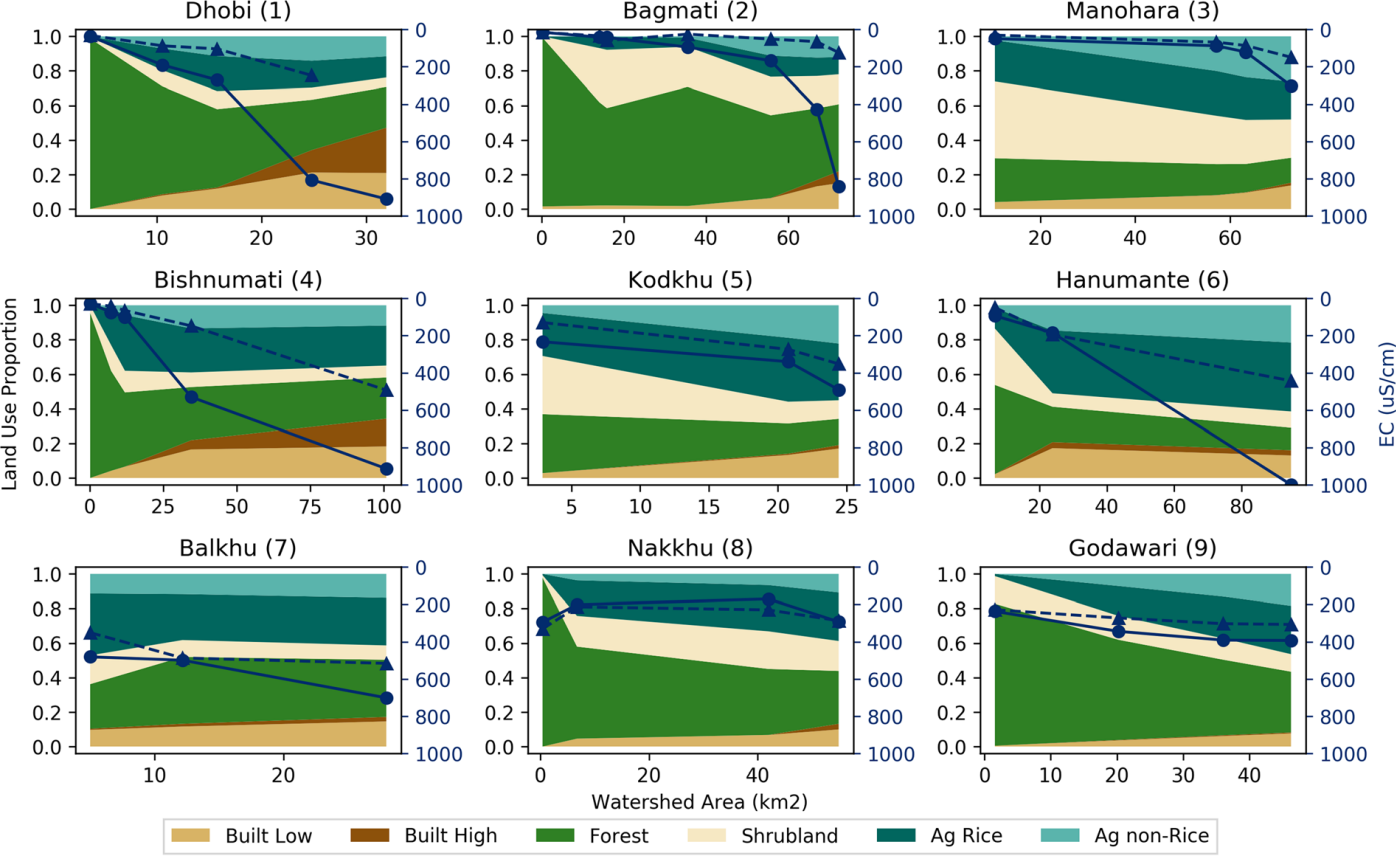
Land-use proportions and electrical conductivity (EC) results for the nine perennial streams in the Kathmandu Valley. Land-use proportions shown for six land-uses classes with reference to the primary (left) vertical axes. EC results in micro siemens per centimeter (μS cm^−1^) shown for 2016 monsoon (dashed line with triangles) and 2017 pre-monsoon (solid line with circles) periods with reference to the secondary (right) vertical axes

**Fig. 8:**
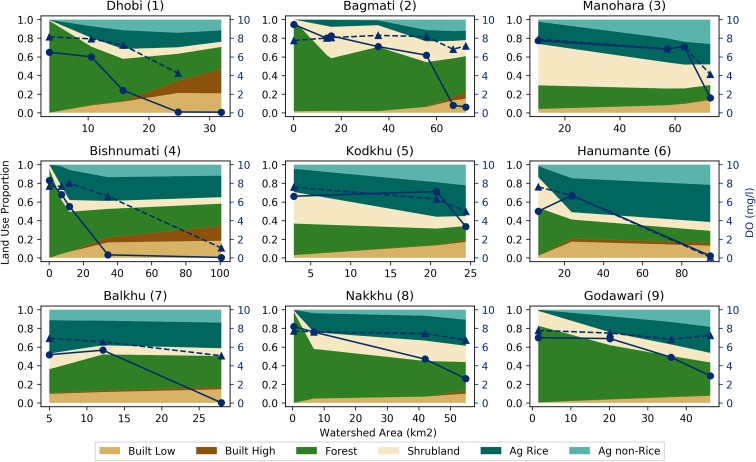
Land-use proportions and dissolved oxygen (DO) results for the nine perennial streams in the Kathmandu Valley. Land-use proportions shown for six land-uses classes with reference to the primary (left) vertical axes. DO results in milligrams per liter (mg l^−1^) shown for 2016 monsoon (dashed line with triangles) and 2017 pre-monsoon (solid line with circles) periods with reference to the secondary (right) vertical axes

**Fig. 9:**
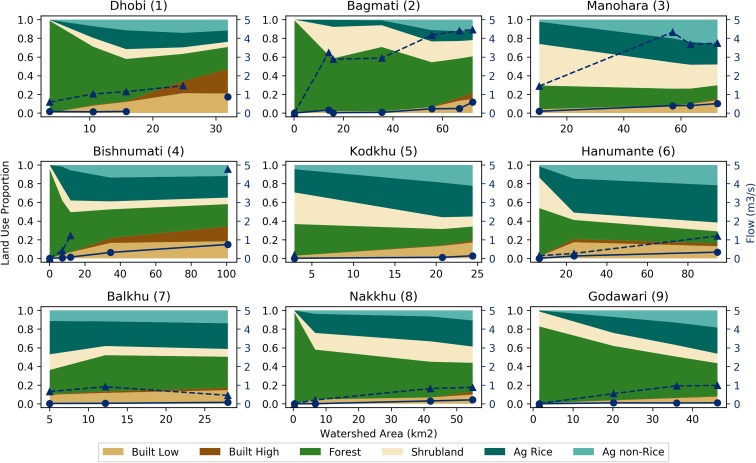
Land-use proportions and flow results for the nine perennial streams in the Kathmandu Valley. Land-use proportions shown for six land-uses classes with reference to the primary (left) vertical axes. Flow results in cubic meters per second (m^3^ s^−1^) shown for 2016 monsoon (dashed line with triangles) and 2017 pre-monsoon (solid line with circles) periods with reference to the secondary (right) vertical axes

Figures 6, 7, 8, and 9 present three by three arrays of the land-use proportion and water quality and flow data for the nine perennial streams in the Kathmandu Valley. The data are presented in the same way as the Bagmati River watershed data (Fig. 5). RQC and EC data are plotted with the secondary (right) vertical axes reversed so that values that move vertically downward on the plot areas represent a decline in ecological stream health or water quality.

RQC had deteriorating trends from upstream to downstream for all nine watersheds (Fig. 6). The steepest declines in ecological stream health occurred in the Dhobi (1), Bishnumati (4), and Hanumante (6) watersheds. These watersheds had the largest and upstream most occurring proportions of built (low and high) uses. A deterioration in ecological stream health from 2016 monsoon to 2017 pre-monsoon periods was observed at the Dhobi (1), Bagmati (2), and Godawari (3) watersheds (Fig. 6). The largest improvement in ecological stream health from monsoon to pre-monsoon was one RQC, while the largest deterioration was two RQC. All watersheds during 2017 pre-monsoon had RQC 5 at the most downstream measurement site. During the 2016 monsoon, the Kodkhu (5), Nakkhu (8), and the Godawari (9) had RQC of 4, 4, and 3, respectively.

Both EC and DO showed similar deteriorating trends from upstream to downstream for all nine watersheds (Figs. 7 and 8). An increase in EC was observed at eight out of the nine watersheds from 2016 monsoon to 2017 pre-monsoon; EC levels decreased slightly at the Nakkhu watershed. The largest changes in EC were observed at the Dhobi (1), Bagmati (2), Bishnumati (4), and Hanumante (6) (Fig. 7). The largest declines in DO were observed at the Dhobi (1), Bagmati (2), Bishnumati (4), and Balkhu (7) watersheds (Fig. 8). During 2017 pre-monsoon, the Dhobi (1), Bagmati (2), Manohara (3), Bishnumati (4), Hanumante (6), and Balkhu (7) watersheds all had DO values below 2 mg l^−1^.

Flows showed increasing trends from upstream to downstream for all watersheds in the 2016 monsoon and 2017 pre-monsoon periods (Fig. 9). All flows during the 2017 pre-monsoon were less than 2016 monsoon. On average, flows during the pre-monsoon were 11% of monsoon values (min = 0.6%; max = 49%; SD = 12%). During the pre-monsoon period, precipitation and runoff are low. Even still, we observed steady increases in streamflow from upstream to downstream, especially in areas with Built Low and High land-uses. Our hypothesis is that this increase in flow is due to wastewater return flows from either surface water or groundwater sources. A subsequent publication will explore the possibility of solving for net groundwater pumping from stream reach water balance analyses in the pre-monsoon period.

### Correlation analyses

Pearson’s *r* values between variables for both 2016 monsoon and 2017 pre-monsoon are shown in Table 2 (*n* = 38, *p* = 0.01, *r* > 0.430). For the 2016 monsoon period, 21 out of the possible 28 correlations (i.e., all except flow) were statistically significant. During the 2017 pre-monsoon period, 24 out of 28 correlations were statistically significant. For both monsoon and pre-monsoon data, RQC had significant correlations with all three land-use groups. RQC had a positive correlation with Built and Ag land-uses, meaning that ecological stream health deteriorated as the proportions of Built and Ag lands increased. In contrast, RQC was negatively correlated with Natural lands. Both temperature and EC increased, while DO decreased, as proportions of built and agricultural land-uses increased. Natural lands had the exact opposite impact. Temperature and EC decreased, while DO increased, as proportions of natural lands increased.

**Table 2:**
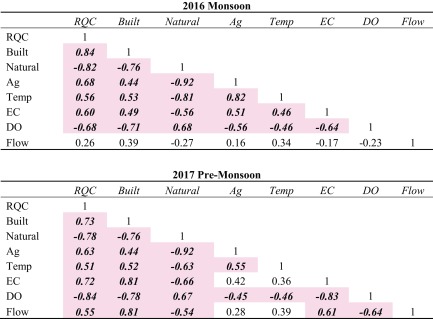
Pearson’s *r* values between RQC, Built (i.e., sum of Built Low and High), Natural (i.e., sum of Forest and Shrubland), Ag (i.e., sum of Ag Rice and Ag Non-Rice), temperature (Temp), electrical conductivity (EC), dissolved oxygen (DO), and streamflow (FLOW). Statistically significant values (*n* = 38, *p* = 0.01, *r* > 0.430) shown in bold italics with a highlighted background

## Conclusions and discussion

Headwater land-uses were dominated by natural (i.e., Forest and Shrublands) land-uses (Figs. 3 and 6). Moving downstream, land-uses transitioned to agriculture (i.e., Ag Rice and Ag Non-Rice) and then to built (high and low density; Figs. 3 and 6). Due mostly to mild topography and a lack of legal protections, some watersheds (i.e., Manohara, Kodkhu, and Balkhu; Fig. 6 (3, 5, and 7) do not have large percentages of natural land-uses upstream of the first RSA monitoring points. This results in the most upstream RQC and water quality values being already impaired. These upstream to downstream land-use trends are a function of topography, historical population areas, and protected areas. The hills surrounding the Kathmandu Valley have steeper slopes than the Valley floor, which constrains agricultural and built land-uses. There are also several protected areas and community forests in the surrounding hills inhibit agricultural and built expansion.

Spatially, RQC increased (i.e., deteriorated in quality) moving radially inward toward the center of the Valley where Built land-uses dominated (Fig. 4). Similar trends for EC and DO were observed (Figs. 7 and 8). Correlation analyses showed that Built land-uses had the strongest impact on RQC, EC, and DO (Table 2).

Temporally, RQC mostly stayed the same or deteriorated from 2016 monsoon to 2017 pre-monsoon (Fig. 6). Deviations from this trend were observed at the middle sites of the Balkhu (7) and Nakkhu (8). In general, DO and EC both deteriorated (i.e., DO decreased and EC increased) from monsoon to pre-monsoon (Figs. 7 and 8). Only the Nakkhu (8) had higher EC values in the 2017 monsoon compared to 2016 pre-monsoon values. Regarding DO, only upstream sites from the Bagmati (2), Bishnumati (4), and Nakkhu (8), and the midstream measurement from the Kodkhu, deviated from this trend with higher DO during 2017 pre-monsoon measurements.

### Discussion

Our results are supported by a previous study of the Bagmati River basin (Shah and Shah 2013). Shah and Shah (2013) indicated that nutrients like chloride and ortho phosphate, and the physicochemical parameters temperature and conductivity increased as rivers flowed through urban areas. Built land-uses include both urban and industrial activities. A lack of water treatment facilities leads to direct discharge of wastewater into streams (Shah and Shah 2013; Milner et al. 2015). The organic and nutrient loads of these wastewaters caused the statistically significant correlations observed (Shah and Shah 2013). In built areas, stressors related to effluents, activities and facilities, and solid wastes mainly contribute to deteriorating water quality of rivers (Shrestha et al. 2008; Shah and Shah 2013). However, certain watersheds (i.e., Manohara, Nakkhu, and Godawari) had natural and agricultural land-uses that persisted at higher relative proportions farther downstream compared to other watersheds within the Valley. This helped to maintain ecological stream health over longer stream reaches (Fig. 6 (3, 8, and 9). Therefore, land-use managers should place higher priority on actively managing and protecting lands within the upstream portions of these tributaries.

For the Balkhu (7), the upstream RQC was 4, whereas all other upstream measurements were RQC 1 or 2. Based on discussions with local residents during field work, many of the springs originating in the higher elevations of the southern portion of the watershed are diverted for water supply. The remaining drainage areas are relatively low in elevation and receive less precipitation in relation to other areas of the Valley because of rain shadow effects. We suggest that these two factors, combined with the low proportion of natural land-uses (Fig. 6 (7)) in the watershed, cause the observed low quality.

Shah and Shah (2013) also found that pre-monsoon is the most critical season for ecological condition of the river. They observed that ecological river quality was worst in pre-monsoon compared to post-monsoon season because the amounts of stressors are similar throughout the year, while discharge dramatically reduces during the pre-monsoon. River stretches flowing through built areas had mainly sludge as river substrates with assemblages of nil to few numbers of highly tolerant macroinvertebrates like red Chiromidae and Syrphidae. Since flows during the pre-monsoon are less than the monsoon (on average 11%; Fig. 9), this trend is likely due to the adage that “dilution is the solution to pollution.”

We suggest that our three different visualization techniques (i.e., map-based, graphical, and correlation matrixes) can be used to “tell the story” in these land-use and water quality data to a wide audience of citizens, scientists, and policy makers. The map-based and graphical approaches for visually presenting the relationships between land-use and water quality and quantity provide a framework for communicating these data. The correlation analyses quantify these relationships in a concise way. Additional work should focus on evaluating the effectiveness of these techniques on crucial stakeholders and decision makers.

### Sources of uncertainty

Two land-use assumptions stated earlier are worth revisiting. First, we assumed that land-use was stationary between the image capture date in October 2015, monsoon monitoring in 2016, and pre-monsoon monitoring in 2017. Because of the 18-month interval between the 2015 image capture date and 2017 pre-monsoon monitoring, it is likely that there were some changes to land-uses during this period. While efforts are currently underway to collect additional land-use observations to create an updated land-use map, the results are not yet available. Therefore, there is currently no way to quantify the extent of the land-use changes within the period of this study. We anticipate, however, that any observed changes will be less than a few percentage points. Second, we assumed that the interannual variations in actual land cover within the Agriculture Rice land-use had a negligible impact on our results. While it is likely that these seasonal changes have an impact on evapotranspiration and soil moisture storage, it is unlikely that stream water quality was affected. This is because rainfall, and therefore recharge and runoff were low during the 2017 pre-monsoon sampling period.

Uncertainty in land-use classification ends up directly propagating to uncertainty in the resulting understanding of the impacts of land-use on water quality and flow. Our pixel-based classification methodology is based on probabilities derived from training, so inherently, there is a chance of misclassification. Our validation process indicated that roughly 88% of the pixels checked were correctly classified. The incorrect classifications were either mistakes between the density (i.e., low or high) of built land-uses, or between the type of agriculture (i.e., rice or non-rice). While built low or high (or agriculture rice or non-rice) are likely to have different impacts on ecological stream health, the magnitude of these differences is poorly understood. To improve confidence in the land-use classification, we suggest that it be updated every 2 to 5 years, and that additional ground truthing observations be performed. This will decrease uncertainty in the classification probabilities, and will increase the size of the validation data set. Despite these uncertainties in land-use classification, we do not observe any systematic biases that would change the primary findings of this investigation.

Additionally, uncertainty in field measurements (e.g., RQC, EC, DO, and Flow) ends up affecting uncertainty in our understanding of the impacts of land-use on water quality and flow. RSA measurements, including (1) sensory, (2) ferro-sulfide reduction, (3) bacteria, fungi, and periphyton, and (4) macroinvertebrate composition observations, are likely the most subjective measurement included in this analysis. Despite the semi-subjective nature of the sensory observations in particular, we suggest that using the same person to perform RSA measurements, as was done for this study, is the best way to remove this source of uncertainty. This is because the same person is most likely to consistently repeat sensory observations using the same standards. EC and DO were measured with a MultiLine® Multi 3630 IDS [WTW Germany] with a stated accuracy of ± 0.5% of the actual value. Based on an ISO discharge uncertainty calculation within the SonTek FlowTracker software, the average uncertainty in flow was 4.9%. Despite these uncertainties in field observations of RQC, EC, DO, and flow we do not observe any systematic biases that would change the main conclusions of this paper.

### Summary

We collected water flow and quality data from 38 locations within the Kathmandu Valley during 2016 monsoon and 2017 pre-monsoon periods. By combining these data with a newly generated land-use coverage, we were able to quantify the impacts of land-use on water quality and flow. There was a statistically significant impact (*p* = 0.01) of land-use on water quality (i.e., RQC, DO, and EC), with built land-uses (both low and high density) having the strongest impact. Our findings emphasize the need to integrate land-use planning and water resource management in general, while specifically underscoring the critically impaired status of the perennial tributaries to the Bagmati River in the Kathmandu Valley.

## Electronic supplementary material

ESM 1 (PNG 1333 kb)

ESM 2 (XLSX 19 kb)
