# Supplementary figures and images for: Quantifying the connections—linkages between land-use and water in the Kathmandu Valley, Nepal
Source: Environ Monit Assess. 2018 Apr 23;190(5):304. doi: 10.1007/s10661-018-6687-2 (PMC5913389; doi:10.1007/s10661-018-6687-2)

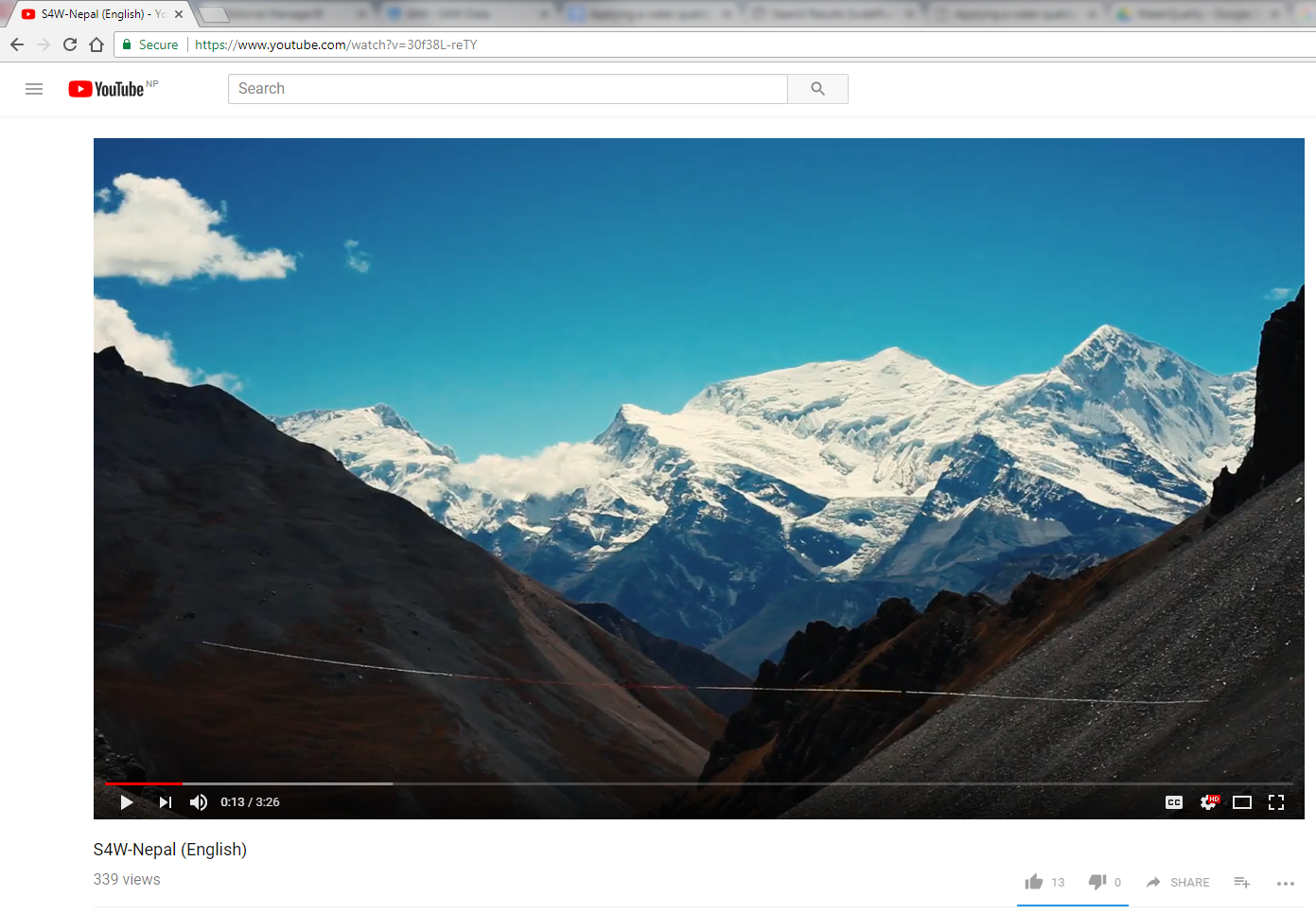

Supplement: Supplementary file 1 — (PNG 1333 kb) [file 10661_2018_6687_MOESM1_ESM.png]
